# A case of Kawasaki disease presenting as sigmoid colitis

**DOI:** 10.1007/s10396-017-0808-3

**Published:** 2017-07-27

**Authors:** Yasuhiro Ohnishi, Kazuhiro Mori, Miki Inoue, Nobuo Satake, Mitsuyasu Yano

**Affiliations:** 1 Department of Pediatrics, Tokushima Prefectural Central Hospital, 1 Chome 10-3 Kuramoto-cho, Tokushima, Tokushima 770-0042 Japan; 2 Department of Medical Gastroenterology, Tokushima Prefectural Central Hospital, Tokushima, Japan; 3 Department of Pathology, Tokushima Prefectural Central Hospital, Tokushima, Japan

**Keywords:** Kawasaki disease, Sigmoid colitis, Ultrasonography, Abdominal pain

## Abstract

Initial gastrointestinal symptoms might confuse the clinical pictures of some patients with Kawasaki disease (KD) and delay diagnosis and treatment, especially when the patient does not fulfill sufficient diagnostic criteria for KD. Here, we present the case of a 4-year-old boy with KD who complained of severe left abdominal pain and fever alone for the first 6 days. Abdominal ultrasonography showed severe wall thickening localized to the sigmoid colon, and these findings were confirmed by computed tomography and colonoscopy. Microscopic examination of a biopsy specimen revealed non-specific colitis with inflammatory cells in the lamina propria of the sigmoid colon, indicating sigmoid colitis. He subsequently exhibited typical symptoms of KD and was successfully treated with oral administration of aspirin. We suggest that KD should be considered as a differential diagnosis in any child presenting with abdominal symptoms and prolonged fever without definable cause. Abdominal ultrasonography can help evaluate the gastrointestinal complications of KD.

## Introduction

Kawasaki disease (KD) is an acute, systemic febrile vasculitis of unknown etiology. Gastrointestinal symptoms including diarrhea and vomiting are relatively common findings. As specific abdominal complications, intestinal pseudo-obstruction [1, 2], hydrops of the gallbladder [3], pancreatitis [4], duodenitis and duodenal perforation [3, 5], and appendicitis [3, 6] have been reported. Gastrointestinal symptoms often obscure the correct diagnosis of KD in cases that do not fulfill sufficient diagnostic criteria for KD. Compared to the involvement of the small intestine, KD with colitis is rather rare. Here, we present the case of a 4-year-old boy who complained of severe left abdominal pain without diarrhea for the first 6 days due to sigmoid colitis. Abdominal ultrasonography proved useful for evaluation of the gastrointestinal complications of KD.

## Case report

A 4-year-old boy was admitted with complaints of severe left lower abdominal pain and high fever (40 °C) for 4 days. Oral antibiotics prescribed by his previous doctor had not proven effective. On admission, he had no symptoms other than left lower abdominal pain and fever. Neither diarrhea nor vomiting was seen during the course. Laboratory examinations revealed: white blood cell count, 23,000/μL; neutrophils, 67%; platelet count, 396,000/μL; and C-reactive protein, 11.7 mg/dL. Other laboratory data were within normal limits. Stool culture demonstrated only nonpathogenic *Escherichia coli*. Abdominal radiography showed a distended bowel loop containing fecal material.

**Fig. 1:**
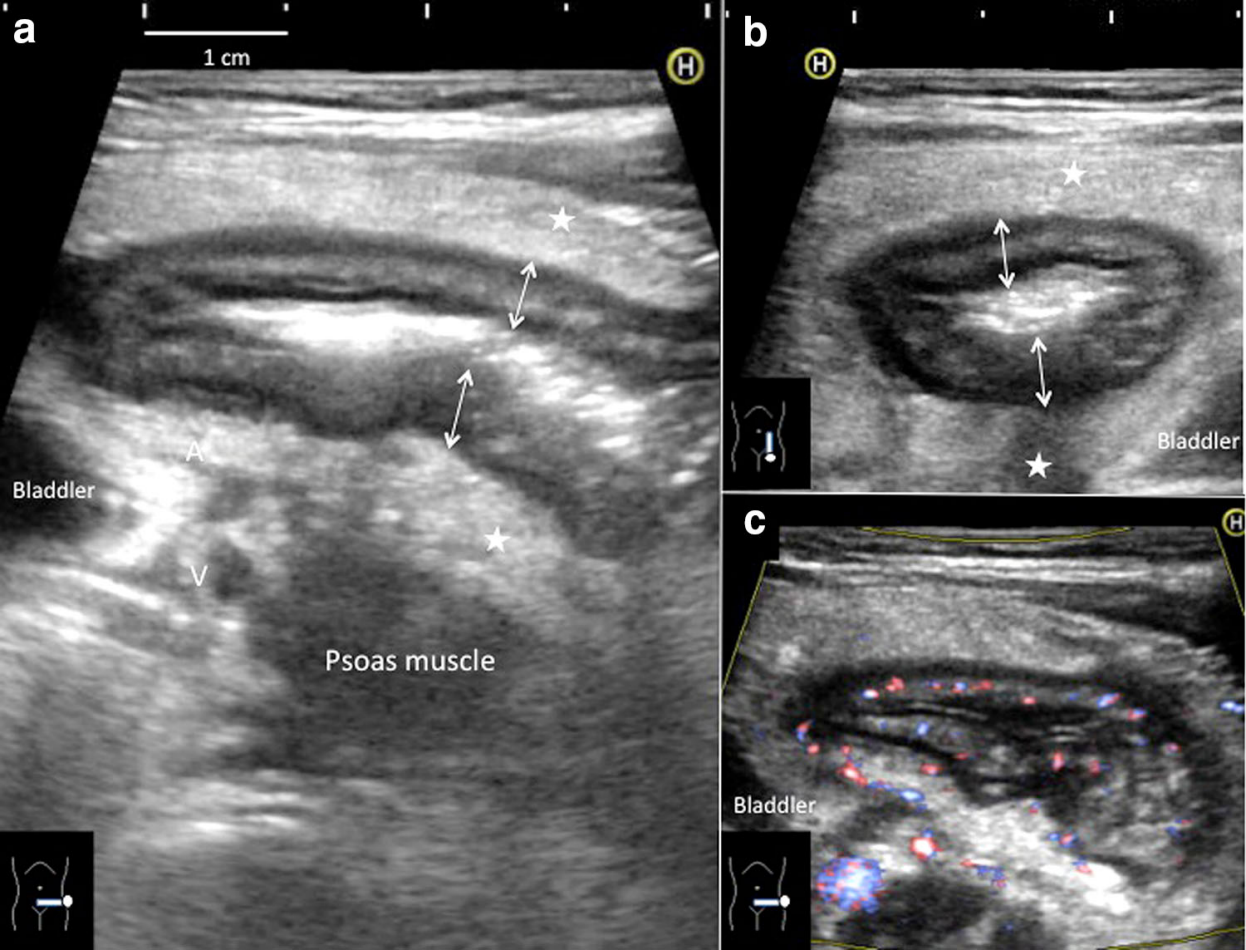
Abdominal ultrasonography on admission. **a** *Transverse view* thickening of the bowel wall to 6 mm (*double-headed arrows*) is recognized in the sigmoid colon, predominantly involving the submucosal layer. Inflammatory changes in the surrounding fat are also prominent (*stars*). **b** *Sagittal view* thickening of the bowel wall is prominent with increased echogenicity of the surrounding fat. **c** *Transverse view* on color Doppler ultrasonography. The color Doppler signal in the sigmoid colon is not increased, which might indicate colonic wall ischemia. *A* iliac artery, *V* iliac vein

Abdominal ultrasonography was performed with linear (3–7 MHz) probes on a HI VISION Avius (Hitachi-Aloka Medical, Tokyo, Japan) and Logic E9 (GE Healthcare, Buckinghamshire, UK). Ultrasonography showed significant thickening of the wall localized to the sigmoid colon, predominantly in the submucosal layer (Fig. 1a, b). Five distinct layers in the bowel wall were recognizable. Inflammatory changes to the surrounding fat were prominent. Color Doppler ultrasonography showed slightly decreased blood flow signals in the sigmoid colon (Fig. 1c).

**Fig. 2:**
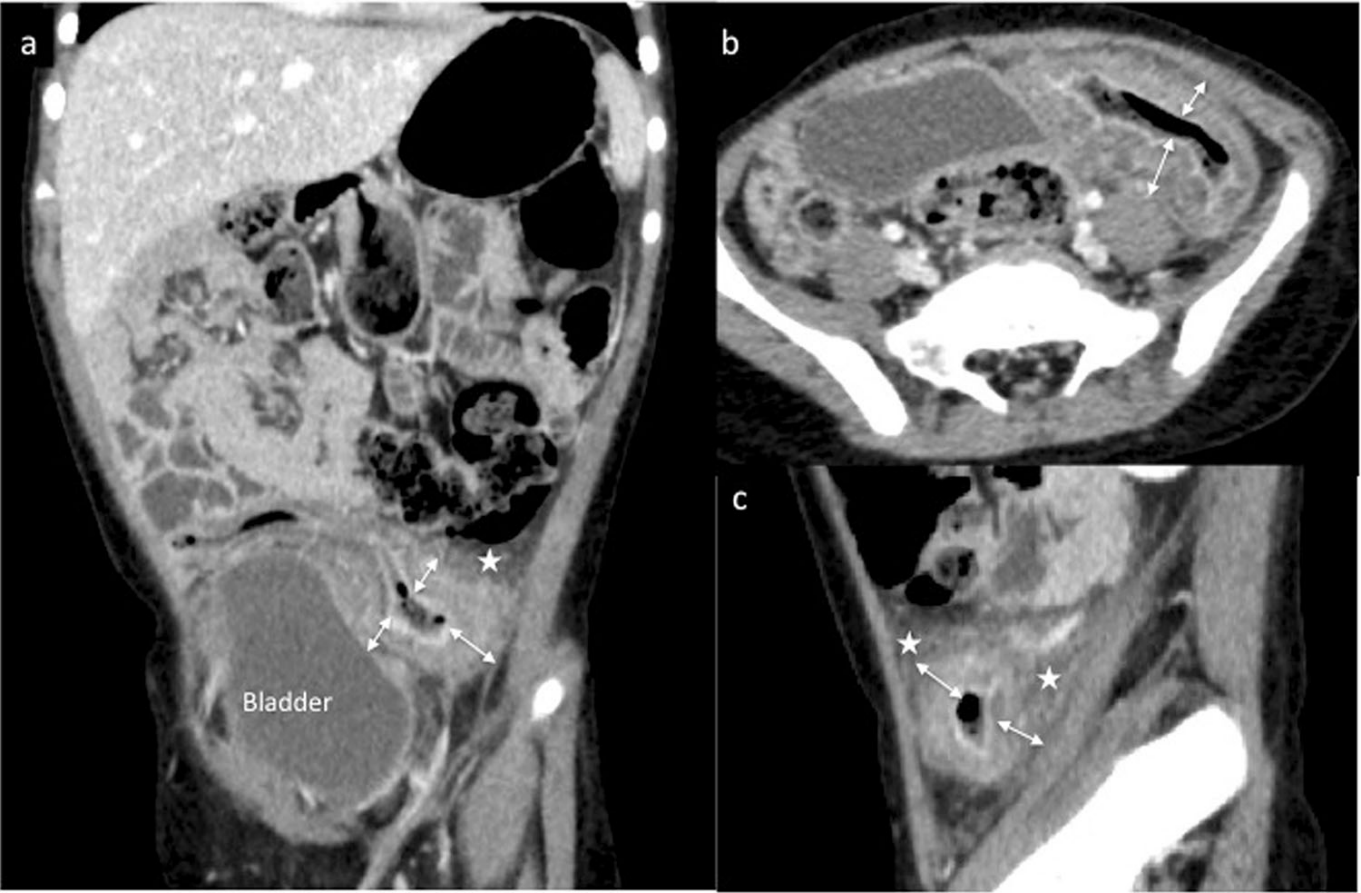
Abdominal CT findings. **a** Coronal view, **b** transverse view, **c** sagittal view. Contrast-enhanced abdominal CT shows thickening of the bowel wall localized to the sigmoid colon (*double-headed arrows*). Inflammation of fat around the sigmoid colon is also apparent (*stars*). The remaining parts of the gastrointestinal tract are almost normal

Contrast-enhanced abdominal CT also revealed localized thickening of the bowel wall in the sigmoid colon (Fig. 2). The remaining parts of the colon showed normal thickness of the wall. No mesenteric ischemia caused by arterial or venous occlusion was detected.

**Fig. 3:**
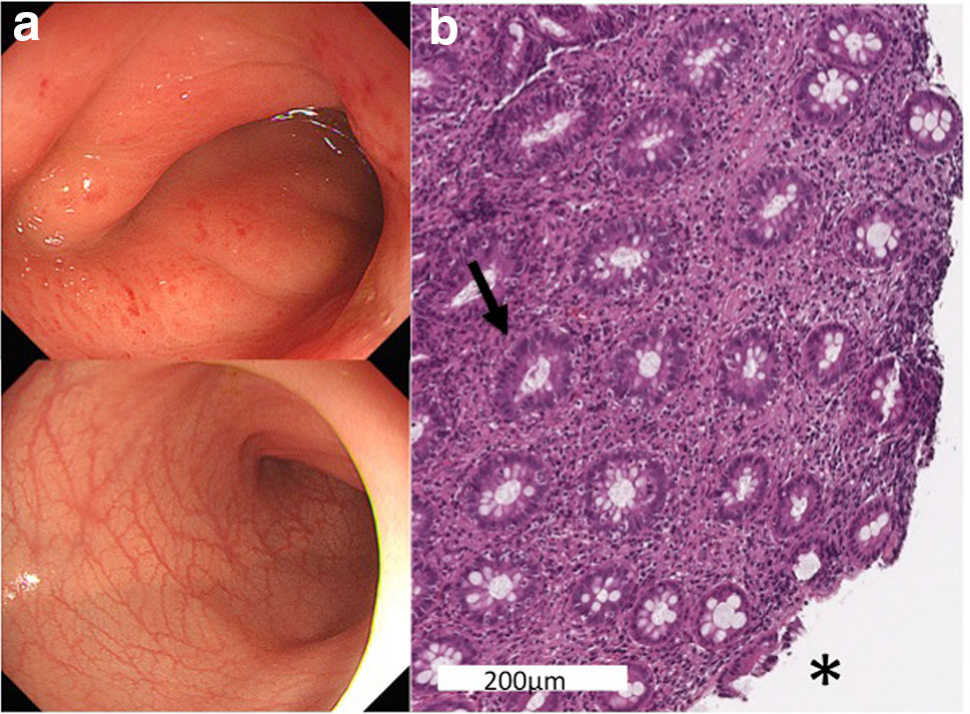
**a** Findings from colonoscopy. *Upper* severe wall-thickening and slight redness of the mucous surfaces are recognized in the sigmoid colon, over a length of 7 cm. The lumen appears narrowed. No bleeding, exudate, ulceration, or findings compatible with inflammatory bowel disease (aphthous ulcer or cobblestoning) are recognizable. *Lower* the remaining colon shows no abnormalities. **b** Microscopic examination of the biopsy specimen. A moderate degree of non-specific cell infiltration by lymphocytes, histiocytes, and neutrophils is evident in the lamina propria (*arrow*). Focal erosion on the mucosal surface is recognized (*asterisk*). No histological findings compatible with idiopathic inflammatory bowel disease are evident

Colonoscopy showed severe circumferential thickening of the bowel wall and slight redness of the mucous membranes localized to the sigmoid colon (Fig. 3a, upper). The lumen of the sigmoid colon was narrowed. No bleeding, exudate, or ulceration was found. Endoscopic findings compatible with inflammatory bowel disease, such as aphthous ulcer or cobblestoning, were not detected. The remaining areas of the colon displayed normal findings (Fig. 3a, lower). Microscopic examination of a specimen from sigmoid colon biopsy revealed non-specific colitis. Inflammatory cells in the lamina propria included lymphocytes, histiocytes, neutrophils, and eosinophils (Fig. 3b). No histological findings indicated idiopathic inflammatory bowel disease, such as crypt abscess, basal plasma cytosis, crypt distortion, and granuloma.

Based on a tentative diagnosis of sigmoid colon colitis, intravenous administration of antibiotics was tried for 2 days. However, both fever and abdominal pain continued. On day 3 after admission, the patient displayed progression of eruptions over the body, swelling of both hands, swollen and cracked lips, strawberry tongue, and bilateral conjunctival hyperemia. At that time, we diagnosed KD and started oral administration of aspirin.

**Fig. 4:**
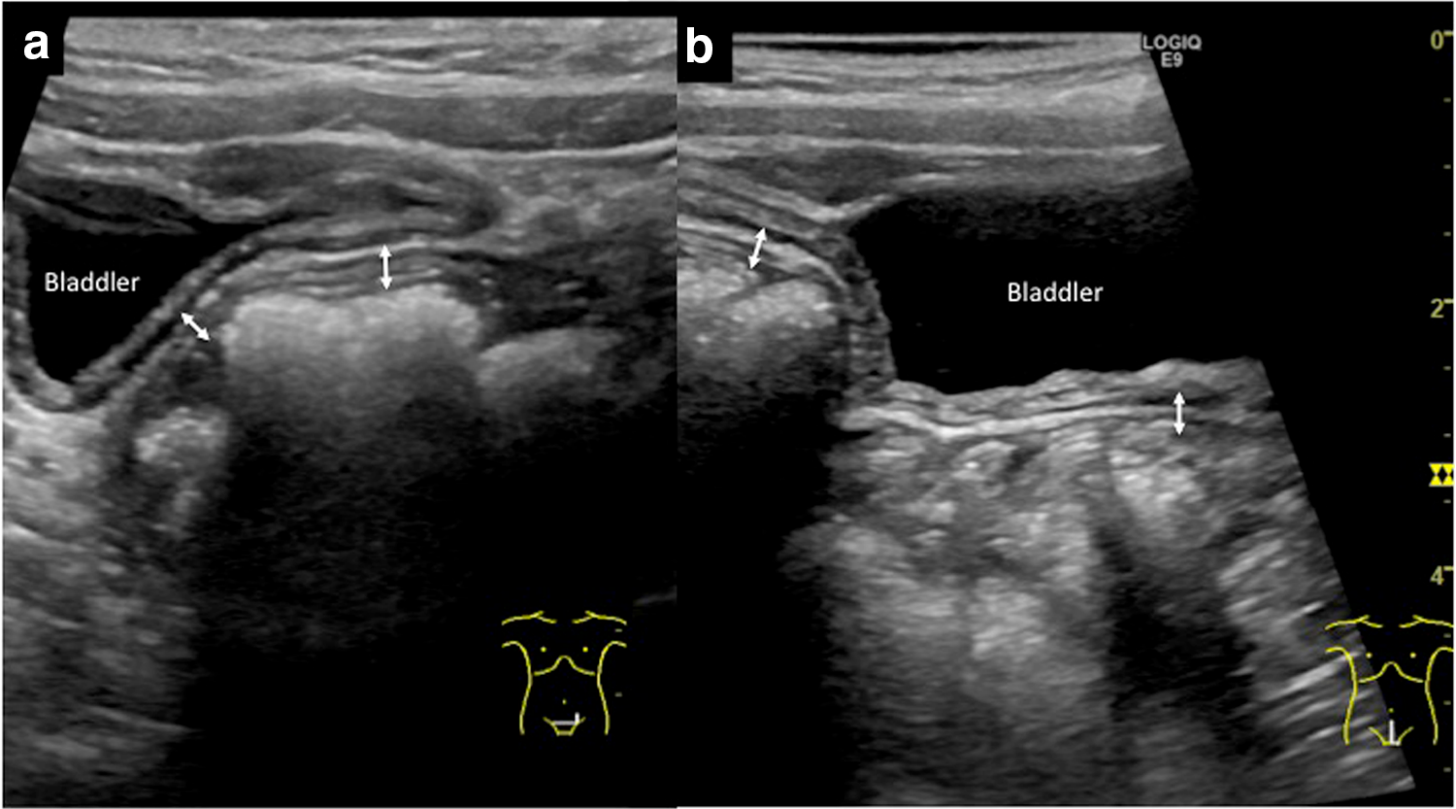
Abdominal ultrasonography at discharge. **a** *Transverse view*, **b** *sagittal view*. In both figures, thickening of the bowel wall in the sigmoid colon has normalized (*double-headed arrows*). Hyperechoic changes to the surrounding fat have also disappeared

After initiating treatment with aspirin, both gastrointestinal symptoms and main symptoms of KD gradually resolved. Ultrasonographic findings of wall thickening in the sigmoid colon also disappeared (Fig. 4). The patient was discharged with low-dose aspirin (3 mg/kg/day) on day 15 after initial symptoms. Peri-ungual desquamation was noted at that time. Repeated echocardiography showed no dilatation of the coronary arteries.

## Discussion

As associated symptoms outside the clinical criteria for KD, gastrointestinal symptoms (vomiting, diarrhea, or abdominal pain) are relatively common. The severity of these symptoms is highly variable among patients. In most cases, gastrointestinal symptoms gradually resolve after the treatment of KD itself. Some case reports have described conservative treatment of small-bowel pseudo-obstruction [1, 2]. On the other hand, emergency abdominal surgery is necessary in some cases of KD with gastrointestinal symptoms. Zulian et al. reported that 10 of 219 patients (4.6%) presented with severe abdominal complaints requiring surgical intervention or endoscopy [3]. The postoperative diagnoses were gallbladder hydrops, paralytic ileus, appendicitis, and hemorrhagic duodenitis. In that study, patients with KD who needed surgical intervention were frequently associated with aneurysms of the coronary arteries (50%). Duodenal perforation has also been reported in a patient presenting with gastrointestinal bleeding, and emergent laparotomy was performed [5]. Garnett described two cases with acute surgical abdomen in whom acute appendicitis was histologically confirmed [6].

If abdominal symptoms are predominant and features typical of KD are not evident, initiation of appropriate medical treatment may be delayed, with potential consequences for the development of cardiac sequelae [3]. Akikusa et al. reported one case presenting with intestinal pseudo-obstruction, and KD was diagnosed only 14 days after the initial onset of symptoms, due to the perception that the patient was primarily suffering from an ‘abdominal’ disease [1]. Maurer et al. suggested that a finding of segmental bowel-wall thickening on abdominal ultrasonography in an acutely ill febrile child should raise the suspicion of KD, and abdominal ultrasonography may help establish the correct diagnosis [7].

Lesions of the sigmoid colon in KD are quite rare among the gastrointestinal complications of KD. To date, only one report has described KD associated with the region of the sigmoid colon. Kim et al. reported the case of a 5-year-old boy with colonic edema localized to the descending and sigmoid colon [8]. In that case, abdominal pain and fever were the main symptoms at disease onset. KD was diagnosed on day 7 after onset. Differential diagnoses for segmental non-specific thickening of the sigmoid colon include idiopathic inflammatory bowel disease and infectious colitis.

Our case showed severe thickening of the submucosal layer of the sigmoid colon. A thickened submucosal layer often indicates an acute process in various bowel diseases and corresponds to either edema or hemorrhage [9]. In KD, this finding is thought to result from edema of the bowel wall due to vasculitis.

No reports have described color Doppler findings in the gastrointestinal tract among patients with KD. Color Doppler signals in the sigmoid colon were not increased in our case. KD involves vasculitis of the middle-sized arteries, and histopathological examinations have demonstrated that the gastrointestinal lesions in KD are localized to the arteries of the subserosa [10]. Ischemia of the subserosal artery in the sigmoid colon might have played a role in the decreased arterial flow in the bowel wall. Beiler et al. reported a case of KD in which the pathological features evident in the surgical specimen were jejunal vasculitis and ischemia [11].

In conclusion, KD should be considered as a differential diagnosis in any child presenting with abdominal symptoms and prolonged fever with no clear cause. Abdominal ultrasonography can help evaluate the gastrointestinal complications of KD.
